# Long-Term Cost-Effectiveness of Severity-Based Triaging for Large Vessel Occlusion Stroke

**DOI:** 10.3389/fneur.2022.871999

**Published:** 2022-05-13

**Authors:** Lan Gao, Marj Moodie, Nawaf Yassi, Stephen M. Davis, Christopher F. Bladin, Karen Smith, Stephen Bernard, Michael Stephenson, Leonid Churilov, Bruce C. V. Campbell, Henry Zhao

**Affiliations:** ^1^Deakin Health Economics, Institute for Health Transformation, Faculty of Health, Deakin University, Geelong, VIC, Australia; ^2^Department of Medicine and Neurology, Melbourne Brain Centre at The Royal Melbourne Hospital, University of Melbourne, Parkville, VIC, Australia; ^3^Population Health and Immunity Division, The Walter and Eliza Hall Institute of Medical Research, Parkville, VIC, Australia; ^4^Ambulance Victoria, Melbourne, VIC, Australia; ^5^Department of Neurology, Faculty of Medicine, Nursing and Health Sciences, Eastern Health and Eastern Health Clinical School, Monash University, Melbourne, VIC, Australia; ^6^Department of Medicine, Austin Health, University of Melbourne, Melbourne, VIC, Australia

**Keywords:** stroke, ACT-FAST, large vessel occlusion, thrombectomy, direct transfer

## Abstract

**Background and Purpose:**

Pre-hospital severity-based triaging using the Ambulance Clinical Triage For Acute Stroke Treatment (ACT-FAST) algorithm has been demonstrated to substantially reduce time to endovascular thrombectomy in Melbourne, Australia. We aimed to model the cost-effectiveness of an ACT-FAST bypass system from the healthcare system perspective.

**Methods:**

A simulation model was developed to estimate the long-term costs and health benefits associated with diagnostic accuracy of the ACT-FAST algorithm. Three-month post stroke functional outcome was projected to the lifetime horizon to estimate the long-term cost-effectiveness between two strategies (ACT-FAST vs. standard care pathways). For ACT-FAST screened true positives (i.e., screened positive and eligible for EVT), a 52 mins time saving was applied unanimously to the onset to arterial time for EVT, while 10 mins delay in thrombolysis was applied for false-positive (i.e., screened positive but was ineligible for EVT) thrombolysis-eligible infarction. Quality-adjusted life year (QALY) was employed as the outcome measure to calculate the incremental cost-effectiveness ratio (ICER) between the ACT-FAST algorithm and the current standard care pathway.

**Results:**

Over the lifetime, ACT-FAST was associated with lower costs (–$45) and greater QALY gains (0.006) compared to the current standard care pathway, resulting in it being the dominant strategy (less costly but more health benefits). Implementing ACT-FAST triaging led to higher proportion of patients received EVT procedure (30 more additional EVT performed per 10,000 patients). The total Net Monetary Benefit from ACT-FAST care estimated at A$0.76 million based on its implementation for a single year.

**Conclusions:**

An ACT-FAST severity-triaging strategy is associated with cost-saving and increased benefits when compared to standard care pathways. Implementing ACT-FAST triaging increased the proportion of patients who received EVT procedure due to more patients arriving at EVT-capable hospitals within the 6-h time window (when imaging selection is less rigorous).

## Introduction

Endovascular thrombectomy (EVT) has been increasingly offered to patients with large vessel occlusion (LVO) stroke. However, the restricted availability of EVT-capable comprehensive stroke centers (CSCs) worldwide has highlighted the importance of correctly and rapidly identifying patients who may benefit from EVT. Delayed EVT is associated with worse functional outcomes (1, 2) and multiple observational studies have linked the delay from secondary inter-hospital transfer with worse outcomes after EVT compared to direct-presenting patients.

Ambulance Clinical Triage for Acute Stroke Treatment (ACT-FAST) is a severity-based screening algorithm administered by paramedics to triage patients who would benefit from direct transfer to a CSC (3). A large real-world validation study showed that ACT-FAST had a sensitivity and specificity of 82.6 and 77.9% respectively in diagnosing LVO for patients with suspected stroke. While the ACT-FAST paramedics validation study reported the time saved from correctly bypassed patients and the avoidance of a potential delay in thrombolysis by bypassing the nearest PSC, the net impact (i.e., both correct and incorrect triaging) on patient outcomes and cost-effectiveness of ACT-FAST remain unclear.

This study aims to translate the ACT-FAST diagnostic accuracies reported in the validation study into long-term health benefits and assess the cost-effectiveness of a severity-based triaging system and estimate potential monetary savings nationally to inform the policy decision-making around its wider implementation.

## Methods

### Study Population

Victorian patients residing in metropolitan area with symptoms suspected of stroke diagnosed by paramedics were simulated. The demographic characteristics of the simulated patients were defined according to a study examining the diagnostic performance of ACT-FAST in Victoria, Australia. Briefly, a total of 522 patients were screened between November 2017 and July 2019 by paramedics from Ambulance Victoria which is the sole service provider for medical emergency transfers in the state of Victoria. Study patients were transferred to 32 hospitals including 15 metropolitan and 17 rural with only the metropolitan hospitals having EVT and neurosurgery capacity (four CSCs in metropolitan Victoria have full-time EVT capabilities). LVO was defined as the intracranial internal carotid artery (ICA), first segment middle cerebral artery (M1-MCA) and basilar artery occlusions. Extended LVO includes complete or near-occlusion of extracranial or intracranial internal carotid, middle cerebral (M1/proximal-to-mid M2), basilar and proximal posterior (P1) cerebral arteries, cerebral artery dissection or symptomatic intracranial atherosclerosis. Diagnostic performance for diagnosing LVO was assessed in the simulation model. Ethical approval was provided by the Royal Melbourne Hospital Research Ethics Committee with a waiver of patient consent.

### Data Availability Statement

Data from the validation study may be requested from the corresponding author in the anonymised form by any qualified investigator.

### ACT-FAST Diagnostic Performance

ACT-FAST involves a two-step examination plus an eligibility and stroke mimic screen step. The former includes (1) unilateral arm drift to stretcher <10 s, (2) severe language deficit (if right arm is weak) or gaze deviation/hemineglect assessed by simple shoulder tap test (if left arm is weak), and (3) eligibility and stroke mimic screen (4). The eligibility and stroke mimic screen are intended to check whether patients meet the criteria for EVT procedure and to exclude patients with prior severe disability/brain tumor/seizures. The details of the algorithm are reported elsewhere (4). The sensitivity and specificity in identifying patients with LVO stroke were informed by the ACT-FAST validation study. It was assumed that LVO stroke constitutes 10% of all strokes. On average, the time to screen patients on the scene was <1 min.

### Simulation Model

**Figure 1:**
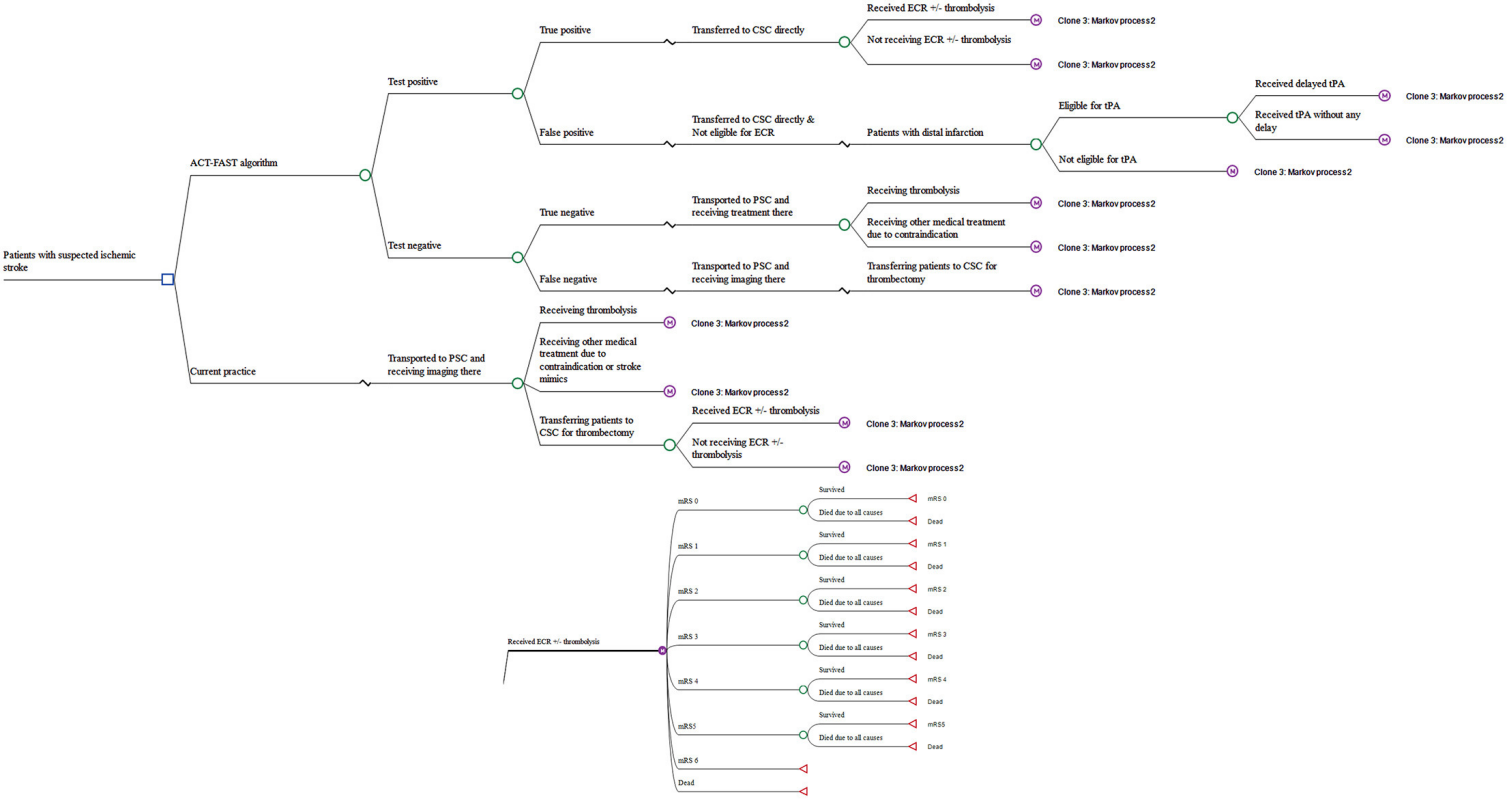
Model structure for the long-term cost-effectiveness analysis. ACT-FAST, Ambulance Clinical Triage for Acute Stroke Treatment; PSC, primary stroke center; CSC, comprehensive stroke center; ECR, endovascular clot retrieval; tPA, tissue plasminogen activator; mRS, modified Rankin scale.

A decision-tree combined with a Markov cohort model was developed to quantify the cost-effectiveness of a severity-based bypass strategy using ACT-FAST. The comparator was the current standard care pathway where all patients are transferred to the nearest thrombolysis center (i.e., primary stroke center, PSC) or CSC, and those requiring EVT who presented to PSC initially may need secondary transfer (i.e., drip and ship model). Patients who received EVT were also considered for intravenous thrombolysis using standard eligibility criteria (Figure 1). In the decision-tree component of the simulation model, the sensitivity and specificity of ACT-FAST screening tool were incorporated to comprehensively assess the variations in the long-term cost-effectiveness. Following either a positive or negative screening outcome at the scene, patients were then classified as true-positive, false-positive, true-negative and false-negative. In this way, the consequences associated with false-positives (i.e., patients without LVO but screened positive) and false-negatives (i.e., patients with LVO but screened negative) were considered and quantified. In addition, the proportion of patients being true-positive (patients with LVO and screened positive) but that did not receive EVT due to other considerations (e.g., established infarct) were also accounted for in the modeled simulation.

For the standard care pathway where ACT-FAST is not available, a proportion of patients in the model were transferred to the PSC initially while CSC was the closest option for the remaining metropolitan patients. Regardless of the capabilities of the hospital where the patient initially presents, an imaging examination will identify a proportion of patients who are eligible for EVT and, among these, a proportion will actually receive EVT. For patients not requiring EVT, thrombolysis or other evidence-based medical treatment will be administered. The proportion of patients who had EVT indicated but were firstly transported to the closest PSC, then require inter-hospital transfer to a CSC for EVT (i.e., a time delay in receiving EVT).

Functional outcomes in ACT-FAST and standard care pathways were determined by the 3-month modified Rankin Scale (mRS) grade with shifts estimated from known outcome-time relationship curves for both thrombolysis and EVT (1, 5). If patients survived the acute event, the age-dependent death rate was applied, adjusted by the hazard ratio (HR) of mortality due to stroke (6). The structure of the simulation model is shown in Figure 1. TreeAge pro healthcare was used for the model-based simulation (TreeAge Pro 2020, R2. TreeAge Software, Williamstown, MA).

### Model Inputs

#### Post-stroke Functional Outcomes

Treatment effectiveness by treatment (EVT ± tPA, tPA, or other medical treatment) was informed by published randomized controlled trials/systematic review of RCTs (1, 5, 7, 8) while adjusted for the time of treatment initiation where applicable. For the ACT-FAST bypass strategy, the time to EVT arterial-access was 52 mins (median, base case) or 59 mins (mean, sensitivity analysis) faster [95% Confidence interval (CI) 40.0–61.5 mins] for all screened true-positive patients who were transferred to a PSC initially (i.e., 60%) (7). For all the ACT-FAST screened false-positives, post-tPA outcomes were adjusted for 10 mins (95%CI 6–12) of treatment delay using the curve from a meta-analysis of tPA trials (7). The effect of a range of delay in receiving tPA (up to 30 mins) was tested in the sensitivity analysis. For patients screened as ACT-FAST negative (either true or false negatives), transfer was modeled to the closest thrombolysis center and post-stroke outcomes were assumed to be the same in both the ACT-FAST and standard care scenarios for these patients, regardless of eventual treatment. Under the ACT-FAST triage system, the percentage of patients receiving thrombolysis at non-CSCs will decrease due to reduction in LVO, so the model was adjusted accordingly for these true-negatives. Meanwhile, for ACT-FAST false-negative patients, no time delay in symptom onset-to-arterial puncture was applied given these patients would follow the same treatment pathway as the controls. The mRS outcomes at 3 months were derived from meta-analyses of RCTs on EVT (1) and tPA (5), respectively.

For patients with LVO stroke, the functional outcomes of both treatments group from EVT trials were applied in the simulation model. The 52 mins delay in symptom onset-to-arterial access time was associated with an absolute risk difference of −7.7% (i.e., mRS shift), this was used to adjust the mRS outcomes of all patients receiving EVT for ACT-FAST triaged patients (5, 8). For patients who received thrombolysis in either scenario, the 3-month mRS outcomes were informed by a meta-analysis of tPA RCTs.

The distribution of the onset age of simulated stroke population was informed by the national statistics (Supplementary Table 1).

#### Costs and Utility Values

The Australian healthcare system perspective was taken to measure the costs related to implementing the ACT-FAST severity-based triaging tool in metropolitan Melbourne in the state of Victoria, Australia. Costs including administering ACT-FAST on the scene (training the paramedics and time consumed for triaging), acute stroke care for the indexed event (9), and post-stroke management (rehabilitation, outpatient care, and nursing home care) (10–12) were considered in the long-term modeled cost-effectiveness analysis. The cost of initial ambulant transfer and any secondary inter-hospital transfer were also incorporated (Ambulance Victoria fee schedule). All the costs were expressed in 2020 Australian dollars (1 AUD = 0.7007 USD, 2020).

The health-related quality of life (utility value) representing each of the Markov states was obtained from published literature.

All the model inputs and their sources are summarized in Table 1. mRS outcomes post 3-month of stroke are presented in Table 2. The illustration of the simulation process is shown in Supplementary Figure 1 and Supplementary Materials.

**Table 1:** Inputs for the simulation model.

**Variable**	**Baseline value**	**Sensitivity analysis (range and distribution^∧^)**	**References**
ACT-FAST diagnostic accuracy			
Sensitivity	0.826	0.70–0.88	(3, 14)^∧^
Specificity	0.779	0.48–0.89	(3, 15)^∧^
Proportion of LVO stroke	0.10	0.10–0.20	(3)
Proportion of patients received EVT at CSC	0.682	0.50–0.80	Assumption
Proportion of distal infarction (false positive) eligible for thrombolysis	0.381	–	(3)
Proportion of patients received thrombolysis in the current practice	0.10	0.05–0.14; Beta distribution (alpha 48.03, beta 432.31)	2017 National Stroke Audit
Proportion of patients being transported for EVT in the current scenario	0.10	0.02–0.15	(16); assumption for the range
Hazard ratio for mortality			(6)
mRS 0	1.53	1.23–1.83; Gamma distribution (alpha 281.70, lambda 184.12)	
mRS 1	1.52	1.20–1.83	
mRS 2	2.17	2.14–2.20	
mRS 3	3.18	3.17–3.19	
mRS 4	4.55	4.31–4.78; Gamma distribution (alpha 4,060.14, lambda 892.34)	
mRS 5	6.55	6.12–6.98; Gamma distribution (alpha 2,513.07, lambda 383.67)	
Utility weight			(17, 18) for the range
mRS 0	1		
mRS 1	0.91	0.869–0.952; Beta distribution (alpha 467.79, beta 46.26)	
mRS 2	0.76	0.723–0.797; Beta distribution (alpha 1,095.91, beta 346.08)	
mRS 3	0.65	0.610–0.689; Beta distribution (alpha 1,025.92, beta 552.42)	
mRS 4	0.33	0.299–0.359	
mRS 5	0	0–0.071	
Cost of ACT-FAST triaging	$12		
Cost of thrombolysis	$3,342	$1,637–3,944	(9)
Cost of thrombectomy	$14,331	$13,131–19,919	(9)
Cost of acute stroke hospitalization	$25,571	$11,238–$32,287	NHCDC (Round 23) (9)
Cost of ambulance transfer	$1,256		Ambulance Victoria
Cost of post-stroke management^*^			(10–12)
Stroke management cost (mRS 0)			
≤1 year	$10,499	$8,399–$12,599	
> 1 year	$1,431	$1,145–$1,717	
Stroke management cost (mRS 1)			
≤1 year	$13,230	$10,584–$15,876	
>1 year	$1,431	$1,145–$1,717	
Stroke management cost (mRS 2)			
≤1 year	$15,943	$12,754–$19,132	
>1 year	$1,814	$1,451–$2,177	
Stroke management cost (mRS 3)			
≤1 year	$17,540	$14,032–$21,048	
>1 year	$1,814	$1,451–$2,177	
Stroke management cost (mRS 4)			
≤1 year	$20,722	$16,618–$24,926	
>1 year	$14,027	$11,222–$16,832	
Stroke management cost (mRS 5)			
≤1 year	$24,169	$19,335–$29,003	
>1 year	$17,943	$14,354–$21,532	

*LVO, large vessel occlusion; mRS, modified Rankin scale; ACT-FAST, Ambulance Clinical Triage For Acute Stroke Treatment; EVT, endovascular thrombectomy; NHCDC, National Hospital Cost Data Collection, Australia*.

∧ *Sensitivity and specificity of other severity-based triaging tools were used to inform the range tested in the sensitivity analysis*.

* *Cost of post-stroke management includes the costs related to outpatient care, rehabilitation, nursing home care. Parameters for distribution were based on assumptions*.

*Hazard ratio was estimated using the general population as a reference group according to Hong and Saver (6)*.

**Table 2:** Modified Rankin scale outcomes at 3 month by treatment type.

	**Current scenario**		**ACT-FAST**									
	**No tPA**	**tPA**	**Transported to CSC**		**True positive**		**False positive**		**True negative**		**False negative**	
			**EVT** **±tPA**	**No EVT** **±tPA**	**EVT**	**No EVT**	**Late tPA**	**tPA**	**No tPA**	**tPA**	**No tPA**	**EVT**
mRS0	0.143	0.197	0.100	0.05	0.107	0.05	0.193	0.197	0.143	0.197	0.143	0.100
mRS1	0.143	0.202	0.169	0.079	0.181	0.079	0.198	0.202	0.143	0.202	0.143	0.169
mRS2	0.143	0.095	0.191	0.136	0.205	0.136	0.096	0.095	0.143	0.095	0.143	0.191
mRS3	0.143	0.131	0.169	0.164	0.181	0.164	0.133	0.131	0.143	0.131	0.143	0.169
mRS4	0.143	0.132	0.156	0.247	0.167	0.247	0.134	0.132	0.143	0.132	0.143	0.156
mRS5	0.143	0.088	0.062	0.135	0.046	0.135	0.089	0.088	0.143	0.088	0.143	0.062
mRS6	0.143	0.156	0.153	0.189	0.113	0.189	0.158	0.156	0.143	0.156	0.143	0.153

*tPA, recombinant tissue plasminogen activator; EVT, endovascular thrombectomy; CSC, comprehensive stroke center; ACT-FAST, Ambulance Clinical Triage For Acute Stroke Treatment*.

*References: tPA and no tPa: Hacke et al. (19)*.

*Stroke mimics: Mandzia et al. (20)*.

*EVT and no EVT: Goyal et al. (1)*.

### Cost-Effectiveness Analysis

A lifetime time horizon with a yearly cycle (i.e., modeled until all patients had died) was chosen to accumulate the costs and benefits associated with ACT-FAST triaging vs. current standard care pathway adjusted by the half-cycle correction. Quality-adjusted life year (QALY) calculated based on the life year (LYs) survived and quality of life of that survival. Costs and benefits were discounted by 3% annually. An incremental cost-effectiveness ratio (ICER) was estimated based on QALY and LY gains to determine the long-term cost-effectiveness of ACT-FAST triaging system only if it was positive. An often-quoted willingness-to-pay per QALY of A$50,000 was adopted as the decision criteria (13).

### Sensitivity Analysis

To examine the robustness of base case results, both one-way deterministic sensitivity analysis and probabilistic sensitivity analysis were undertaken. For the one-way deterministic sensitivity analysis, screening performance of ACT-FAST (i.e., sensitivity and specificity), proportion of LVO stroke, HR of mortality after stroke, utility weights by mRS score, costs of transfer and thrombectomy procedure, time horizon, and discount rate were tested (Table 1). In addition, the variation in time saved from stroke onset to EVT time (i.e., mean of 59 mins and 95%CI 40.0–61.5 mins) by ACT-FAST and minimal time saved (i.e., 30 and 10 mins) were tested in the sensitivity analysis (2). Moreover, the diagnostic performance of other triaging tools (RACE, LAMS, C-STAT) derived from paramedics validations studies was examined in the current simulation model (14, 15, 21).

For the probabilistic sensitivity analysis, distribution of key drivers of ICER identified from deterministic sensitivity analysis were incorporated (additional methods in Supplementary Material). A total of 5,000 parameters were drawn from each distribution to run through the simulation model and the average of these 5,000 iterations plus 95% confidence interval (CI) were reported. The probabilistic sensitivity analysis assumed that all the distributions were independent (i.e., the variation in one variable does not correlate with another).

Further, the diagnostic accuracy of ACT-FAST algorithm for an extended definition of LVO, reflecting those with evolving EVT eligibility, was examined in the sensitivity analysis. Diagnostic parameters used were a LVO prevalence of 15% and specificity (81.8 vs. 77.9%) was improved at the expense of lower sensitivity (75.8 vs. 82.6%).

### Exploration of National Impact

Impact of the widespread application of the ACT-FAST screening algorithm across Australia was examined. The total number of strokes in Australia from 2019, together with the proportion of LVO strokes in metropolitan area were used to estimate the possible costs and benefits at the national level, excluding two regions (Tasmania and Northern Territory) that transfer interstate to access EVT. The possible point estimate of the weighted QALY gains from the simulation model was multiplied with the size of national population to gauge the potential costs and QALY gains across Australia. The Net Monetary Benefit from implementing ACT-FAST triaging was estimated.

## Results

### Population Characteristics

All 517 patients from the ACT-FAST paramedic validation study were used to define a hypothetical cohort (*N* = 10,000) in this simulation analysis. The average age of patients was 72.3 year (standard deviation, SD 15.6) and 50.2% were males. A total of 168 (32.5%) patients were screened ACT-FAST positive. Baseline brain imaging identified 92/517 (17.8%) LVO strokes using the standard LVO definition. Of 74 patients who underwent EVT, 68 cases were in the metropolitan region and 42/74 (56.8%) patients received secondary EVT transfer. All the patients (including regional and metropolitan) were included for diagnostic accuracy measurement of ACT-FAST however the EVT time saving which was applied in the simulation modeling were solely based on metropolitan patients.

### Cost-Effectiveness Analysis

The mRS score across all the simulated patients at 3 months for ACT-FAST vs. the current standard care pathway showed that a similar proportion of patients in the ACT-FAST scenario (70.75 vs. 70.24%) achieved the functional independence (i.e. mRS ≤ 2).

Over the lifetime, ACT-FAST was associated with lower costs (–$45) and greater QALY gains (0.006) compared to the current standard care pathway, resulting in it being the dominant strategy (less costly but more health benefits). Due to a small proportion of extra patients arriving at EVT-capable centers within the EVT time window from ACT-FAST bypass strategy (who would otherwise miss the EVT treatment window), there would be additional EVT procedure performed. Our simulation estimated that implementing ACT-FAST triaging led to higher proportion of patients received EVT procedures (30 more additional EVT performed per 10,000 patients) (Table 3).

**Table 3:** Results of base case cost-effectiveness analysis.

	**ACT-FAST**	**Control**	**Difference**
Total costs (AUD)	$29,726	$29,770	–*$45*
Cost of acute hospitalization^*^	$14,301	$14,302	–*$1*
Cost of management	$14,136	$14,138	–*$2*
Cost of triaging	$12	$0	*$12*
Cost of ambulance transfer	$1,277	$1,330	–*$53*
Total QALYs	5.005	4.999	*0.006*
Total LYs	5.252	5.248	*0.004*
Number of EVT procedure	0.072	0.069	*0.003*
3-month functional outcome			
mRS ≤ 2	70,748 (70.75%)	70,241 (70.24%)	
mRS>2	29,252 (29.25%)	29,759 (29.76%)	
**Probabilistic sensitivity analysis^∧^**
Total Costs	$29,750 (29,402, 30,092)	$29,786 (29,429, 20,143)	–*$36.14 (−6.36, −61.18)*
Total QALYs	5.013 (4.791, 5.235)	5.005 (4,786, 5.230)	*0.008 (0.005, 0.011)*
Total LYs	5.261 (5.035, 5.488)	5.254 (5.030, 5.484)	*0.006 (0.005, 0.012)*

*AUD, Australian dollar; QALY, quality-adjusted life year; LY, life year; ACT-FAST, Ambulance Clinical Triage for Acute Stroke; mRS, modified Rankin scale; EVT, thrombectomy*.

* *Cost of acute hospitalization includes the costs related to the index hospitalization itself, thrombolysis, and endovascular thrombectomy where applicable. The slight difference in cost of acute hospitalization reflects the small difference in the proportion of patients received EVT and/or thrombolysis*.

∧ *ICER was -$3,948 (95%CI: –$10,782 to –$776). Italic means between-group difference*.

### Sensitivity Analysis

**Figure 2:**
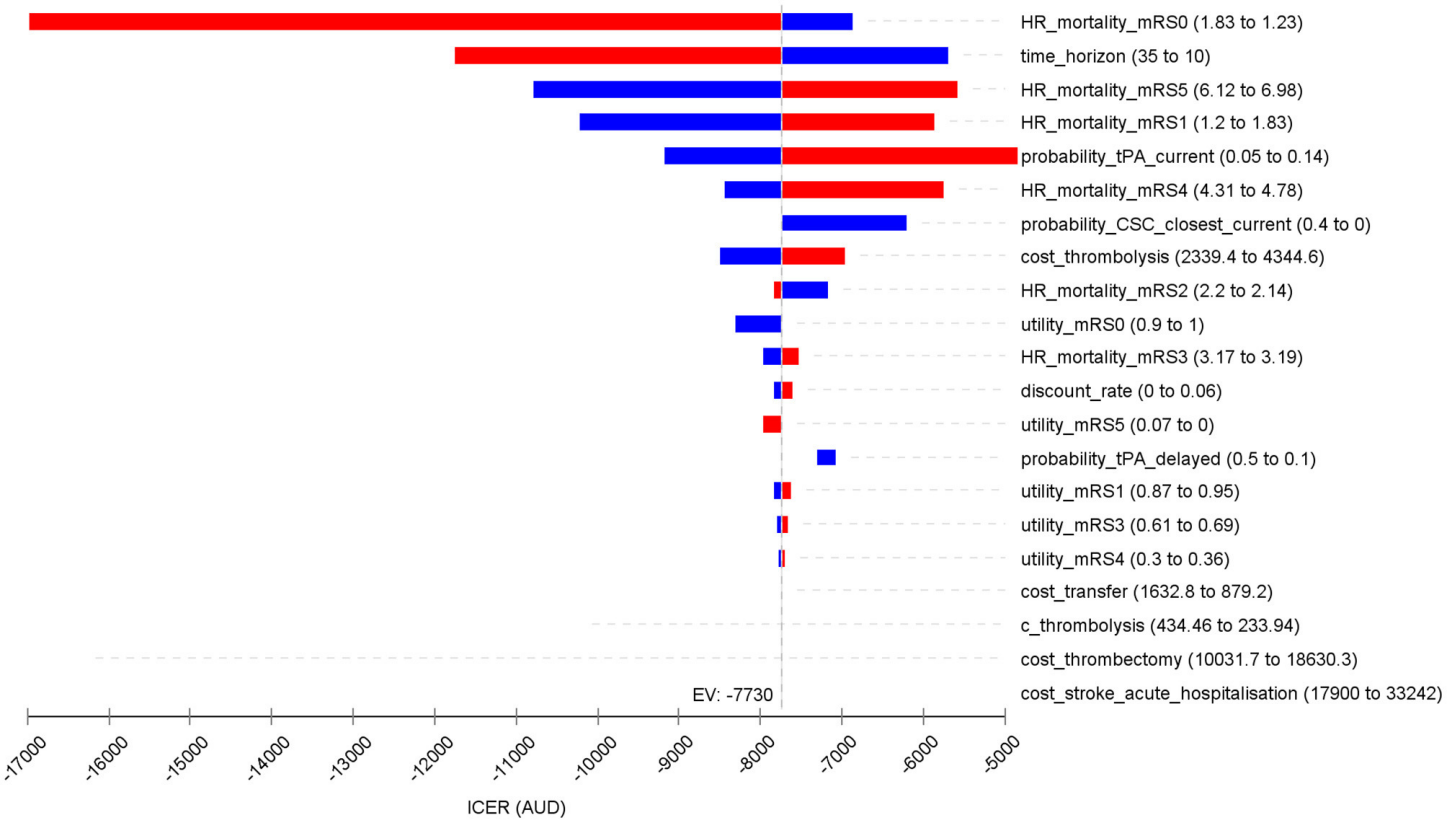
Tornado diagram for the one-way sensitivity analysis showing the variation in the basecase cost-effectiveness results. Slight difference in the ICER (EV) with the abstract and Table 3 (incremental QALY 0.0058 and cost saving of $44.76 per patient) was due to different decimal points.

The one-way deterministic sensitivity analysis indicated that base-case ICER was sensitive to the sensitivity and specificity of ACT-FAST, hazard ratio for mortality post stroke defined by the mRS scores and time horizon (Figure 2). On the other hand, base case ICER were less sensitive to the utility weights associated with each mRS score post index stroke, and costs relating to thrombectomy and hospital transfer.

**Figure 3:**
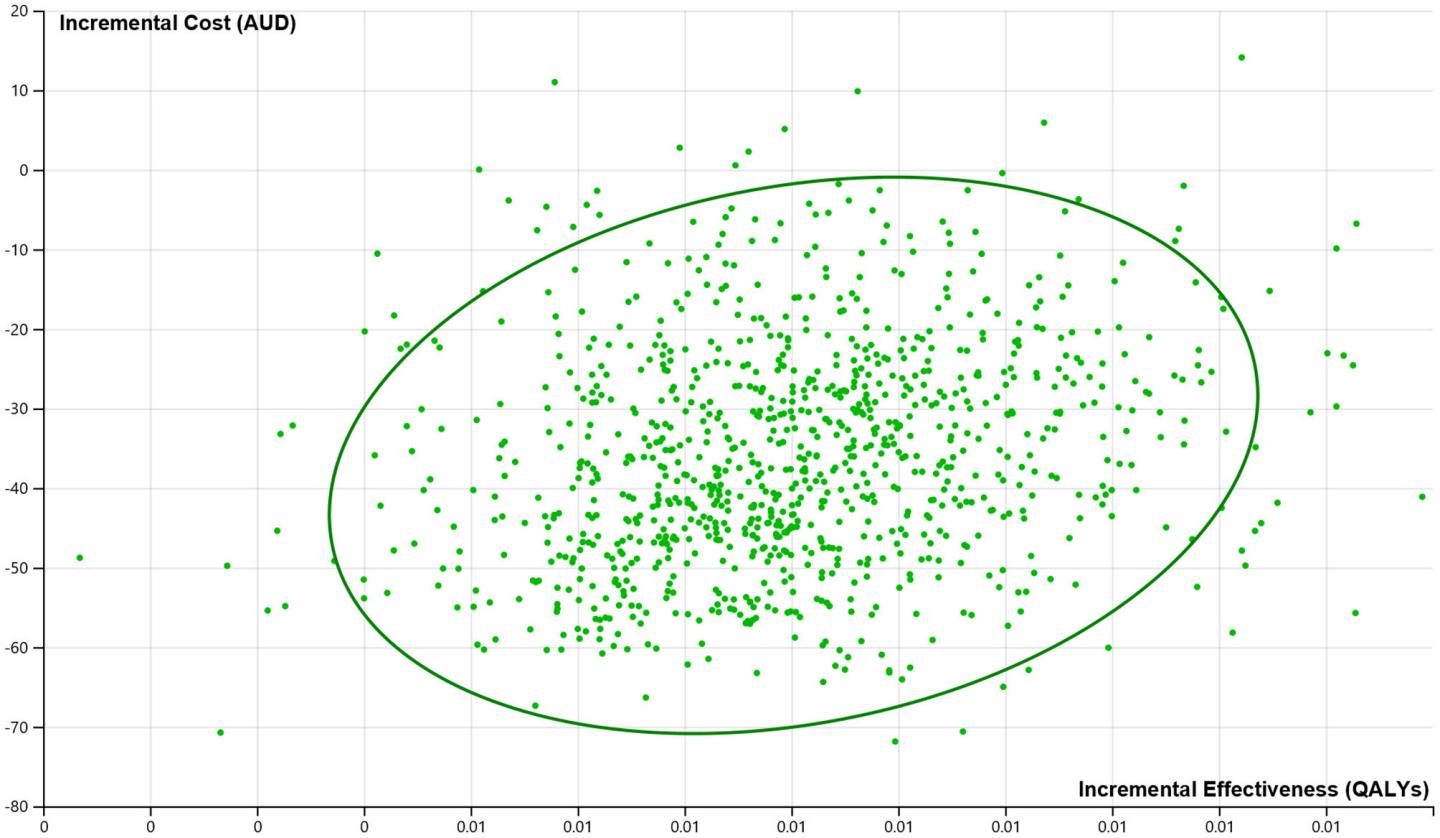
Cost-effectiveness plane from the probabilistic sensitivity analysis indicating the probability of cost-saving and greater effectiveness from ACT-FAST.

The probabilistic sensitivity analysis suggested that ACT-FAST bypass strategy had 99.1% probability of being dominant in triaging suspected stroke patients, indicating lower costs and higher benefits (Figure 3).

Moreover, comparing with other triaging tools (i.e., CPSSS, LAMS, and C-STAT), ACT-FAST bypass strategy remained to be optimal in terms of greater benefits and/or lower costs (Supplementary Table 2). Even if the time saved for EVT commencement from such bypass strategy was reduced to 40 mins, it was still better than the current usual care pathway. The results using average time saved (59 mins) for EVT are highly consistent with the base case results (Supplementary Table 3).

Results using the sensitivity and specificity for an extended definition of LVO were further improved than those from the base case where the LVO definition was employed. The results from this sensitivity analysis are shown in Supplementary Table 2.

### National Impact of ACT-FAST

Based on the number of hospitalisations in Australia due to stroke in 2016–2017 (*n* = 38,055) (22) then excluding patients with non-ischemic and non-LVO stroke, it was estimated that 4,567 patients may be eligible for EVT treatment at baseline in a single year. Assuming that a similar proportion of LVO would benefit from a bypass strategy (i.e., ~50% of LVO patients were from metropolitan areas) from these key states/territories (i.e., accounting for over 96.95% of all strokes nationwide), extrapolating the per patient cost saving and QALY gained would result in a maximum lifetime of A$99,623 cost saving and 13 QALYs gained. Based on a willingness-to-pay threshold of A$50,000 per QALY (23), the total Net Monetary Benefit from ACT-FAST care was estimated at A$0.76 million based on its implementation for a single year.

## Discussion

This study estimated the long-term cost-effectiveness of implementing a severity-based triage strategy using the validated two step examination ACT-FAST algorithm. To our knowledge, this is the first study to translate the diagnostic performance of severity-based triaging into long-term health and cost-effectiveness outcomes. The study synthesizes the sensitivity and specificity of ACT-FAST triaging algorithm to provide a comprehensive examination of its clinical impact and supports its wider implementation. Factoring in both earlier EVT in true-positive cases and potentially delayed thrombolysis in false-positive cases, the use of an ACT-FAST system was associated with an average saving of A$45 and 0.006 QALY gains per patient screened in comparison to current standard care. There are potential health economic savings from the implementation of an ACT-FAST bypass system in Australia, with translated net monetary benefit of A$0.76 million from stroke patients in 2017.

It is worth noting that the ACT-FAST algorithm missed a small proportion of patients (1.7%) with LVO stroke in the simulation study based on its diagnostic performance. However, for the purposes of this study, these missed cases do not affect the cost-effectiveness as their management would be the same regardless of whether an ACT-FAST triage system was in place.

Other severity-based triage tools are available to identify patients with LVO stroke (24). A systematic review was conducted of pre-hospital triage tools/stroke scales in the ambulance service setting. The six tools evaluated—namely NIHSS (≥12), Cincinnati Prehospital Stroke Severity Scale (CPSSS), Prehospital Acute Stroke Severity (PASS), The Los Angeles Motor Scale (LAMS), the rapid arterial occlusion evaluation (RACE) scale, and The Field Assessment Stroke Triage for Emergency Destination (FAST-ED)—had varied sensitivity and specificity. For example, for PASS, LAMS, RACE and FAST-ED, the diagnostic accuracies were similar ranging from 55 to 64% for sensitivity and 83 to 89% for specificity. ACT-FAST had a sensitivity of 82.6% and specificity of 77.9% when assessed in-field by paramedics but heterogeneity between study populations prevents a direct comparison of diagnostic performance. The disability outcomes using other severity-based triage tools may nonetheless be lower than those reported using ACT-FAST triage, particularly if specificity for LVO detection is lower, given the strong influence of specificity in the one-way sensitivity analysis. The sensitivity analyses by adopting the diagnostics performance from LAMS, CPSSS, and C-STAT based on the same simulation model also supported better cost-effectiveness of ACT-FAST.

Preliminary results of the RACECAT randomized trial of prehospital severity-based triage have only been presented in oral form and, overall, did not support the bypass strategy in their locality. However, full analyses with adjustments for imbalances in baseline variables are still to be completed. Furthermore, the study included patients with considerably longer transfers and therefore potential thrombolysis delays was greater than that being applied in metropolitan environments (25). Meanwhile, the validation study of ACT-FAST algorithm was based largely on patients from metropolitan areas in Melbourne, where the significant time saving for earlier EVT far exceeds the minimal delay in thrombolysis. It is considered the results from this cost-effectiveness analysis are still applicable to the local jurisdiction.

This study has some limitations. First, the assumptions for functional outcomes were derived from time savings reported in the ACT-FAST validation study which may be only applicable to metropolitan Melbourne and the uniform time saved (i.e., 52 mins) by ACT-FAST was applied, whereas it may vary in reality according to patients' location. However, we tested the variation in the time saved for EVT and showed that with a minimal of 10 mins saving in time, the triaging system could still be marginally better, which bears implication for rural patients where longer transport is expected. Second, the cost-effectiveness was estimated based on the 3-month mRS outcome post-stroke without consideration of any further improvement in functional status past this timepoint. Third, long-term recurrent stroke was not considered in the simulation. However, as patients with more disabling stroke are more likely to experience a recurrent stroke in the long-term, this may underestimate the benefit provided by a triage strategy. Thirdly, a further potential limitation is that the delay in thrombolysis for patients who received EVT after bypass (i.e., ACT-FAST screened true-positive) was not modeled due to a lack of data. However, recent trials have not shown a major difference in outcome when alteplase was omitted entirely prior to thrombectomy (26–30), suggesting that a small delay in thrombolysis prior to thrombectomy is unlikely to have a major impact on outcomes. Fourthly, there are uncertainties around the estimation of ACT-FAST triaging's national impact based on a single center study. However, the national gain was estimated for only 2,214 LVO patients from non-metro areas in Australia for a single year, which we considered conservative. Lastly, for patients being transported to CSCs while not eligible for EVT, the intangible costs associated with being temporarily away from home, and costs of relatively long-distance transfer (i.e., two-way, comparing to the closest PSCs), and travel time of family members have not been examined in the simulation since the healthcare system perspective was taken (travel cost are non-healthcare). The simulated results indicated that the avoidance of secondary transfer would generally result in a resource saving for ambulance services (i.e., lower costs for ambulance transfer and reduced demand and improved availability of paramedics. True-positive patients may utilize less costly non-emergency ambulance transport back to the PSC afterwards). However, it is acknowledged that there are a proportion of bypassed patients who are not appropriate for CSC or do not eventually receive EVT. The additional initial transport time incurred by bypass in metropolitan areas (median 10 min in Melbourne) is unlikely to have an impact on ambulances resources. However, this would be a relevant consideration if ACT-FAST bypass was to be considered in rural areas. Further, while using an ACT-FAST triage system may result in a small proportion of false-positive patients receiving unnecessary bypass (i.e., CSC overburdening), our validation study estimated that only an additional 1.1 patients/week would be delivered to each CSC who would not require EVT. This small increase is considered manageable with the current workforce in a publicly funded healthcare system. However, in a private health service, it may translate into additional staffing/hospital bed costs. Although our simulation study showed a potential economic benefit of ACT-FAST triaging in rural patients from bypassing the local tPA-capable hospital in Victoria, Australia, it may not be generalisable to all settings and is likely to depend on distance to the CSC, local workflow speeds and geographic conditions. The RACECAT trial in rural and regional Catalonia, Spain (currently unpublished) has been presented as having no overall benefit in using severity-based triage, although the systems of care in the control group may potentially be quite different to that in Australia. As such, we acknowledge that a bypass strategy may not be a universal solution for patients in rural and remote regions.

## Conclusions

Using a severity-based triage system using the ACT-FAST algorithm to bypass likely EVT candidates to a comprehensive stroke center is associated with cost-saving and greater health benefits. The total net monetary benefit was estimated to be A$0.76 million per annum. Wider implementation of an ACT-FAST triage system is therefore expected to reduce avoidable stroke-related disability and result in long-term cost savings.

## Data Availability Statement

The raw data supporting the conclusions of this article will be made available by the authors upon reasonable request.

## Ethics Statement

The studies involving human participants were reviewed and approved by Royal Melbourne Hospital Research Ethics Committee. Written informed consent for participation was not required for this study in accordance with the national legislation and the institutional requirements.

## Author Contributions

LG, MM, BC, and HZ designed and conceptualised the study. LG analysed the data and drafted the manuscript. LC interpreted the data and contributed to the data analysis. NY, SD, CB, KS, SB, MS, LC, BC, and HZ contributed to the data acquisition, interpreted the data, and revised the manuscript. All authors contributed to the article and approved the submitted version.

## Conflict of Interest

The authors declare that the research was conducted in the absence of any commercial or financial relationships that could be construed as a potential conflict of interest.

## Publisher's Note

All claims expressed in this article are solely those of the authors and do not necessarily represent those of their affiliated organizations, or those of the publisher, the editors and the reviewers. Any product that may be evaluated in this article, or claim that may be made by its manufacturer, is not guaranteed or endorsed by the publisher.
