# Supplementary material for: Long-Term Cost-Effectiveness of Severity-Based Triaging for Large Vessel Occlusion Stroke
Source: Front Neurol. 2022 May 13;13:871999. doi: 10.3389/fneur.2022.871999 (PMC9136079; doi:10.3389/fneur.2022.871999)
Supplement: Supplementary file 1 [file Data_Sheet_1.PDF]

Supplementary Materials

Expanded methods (Illustration of calculation)

	**LVO definition**			**Extended LVO definition**		
	**Input**	**Life expectancy**	**Years with disability**	**Input**	**Life expectancy**	**Years with disability**
Sensitivity	82.6%			75.8%		
Specificity	77.9%			81.8%		
Prevalence of LVO stroke	10.0%			15.0%		
Test positive (%)	28.2%	6.556	0.1411	26.8%	6.400	0.1766
True positive (%)	29.3%	*5.709*	*0.3342*	42.4%	*5.709*	*0.3342*
False positive (%)	70.7%	*6.908*	*0.0609*	57.6%	*6.908*	*0.0609*
Test negative (%)	71.9%	5.614	0.3258	73.2%	5.615	0.3247
True negative (%)	97.6%	*5.614*	*0.3269*	95.0%	*5.614*	*0.3269*
False negative (%)	2.4%	*5.635*	*0.2818*	5.0%	*5.635*	*0.2818*

Note: the life expectancy and years with disability presented in the table are based on patients with stroke onset age of 80.

Formulas for the diagnostic performance calculation

Test positive: ((prevalence*sensitivity)+((1-prevalence)*(1-specificity)))

Test negative: ((prevalence*(1-sensitivity))+((1-prevalence)*specificity))

True positive: (prevalence*sensitivity)/((prevalence*sensitivity)+((1-prevalence)*(1-specificity)))

False positive: ((1-prevalence)*(1-specificity))/((prevalence*sensitivity)+((1-prevalence)*(1-specificity)))

True negative: ((1-prevalence)*specificity)/((prevalence*(1-sensitivity))+((1-prevalence)*specificity))

False negative: (prevalence*(1-sensitivity))/((prevalence*(1-sensitivity))+((1-prevalence)*specificity))

With the restricted LVO definition, 28.2% of patients would test positive while 8.26% of being true positive and 19.89% being false positive. In comparison, using the extended LVO definition, 26.8% of patients would test positive including 11.37% of true positive and 15.47% of false positive.

Costs and utilities adjustments with misclassification by ACT-FAST triaging

In the current analysis, false-positive patients who had thrombolysis had their utility (i.e. decreased effectiveness of tPA leading to the reduced QALY gained) modelled to reflect the delay in thrombolysis time. The non-thrombolysed patients had no adjustment in utility as there were no additional transport resources or health outcomes affected in this group. False-negative patients had no adjustment in utility as this is exactly the same as what would occur under the "control" scenario (transferred to the nearest PSC/CSC).

Justifications for the discount rate

The discount rate for both costs and benefits was set at 3% per annum since the ACT-FAST triaging algorithm is associated with higher costs at the beginning while it is expected to produce long-term cost savings and health benefits. The application of a higher discount rate will result in less favorable outcomes than that for an intervention with the same costs, but the health outcomes occur closer to the time the intervention is delivered.

Probabilistic sensitivity analysis

The key variables identified from deterministic sensitivity analyses were tested in the probabilistic sensitivity analysis. In particular, distributions of these variables were constructed according to the characteristics (e.g. beta distribution for utility weights and probabilities, gamma distribution for HR (non-negative values), and Dirichlet distribution for 3-month mRS outcome for true positive patients. A random parameter was drawn from individual distributions to run through the model for each simulated patient (i.e., 10,000 patients). This process was iterated 5,000 times (10,000 patients × 5000 iterations) to generate the results for probabilistic sensitivity analyses.

Online Tables

Table I. Stroke hospitalization in Australia by age and sex in 2016-17

	**Hospitalizations per 100,000 population**		
**Age group (years)**	**Males**	**Females**	**Persons**
<25	8	10	9
25–34	23	29	26
35–44	66	64	65
45–54	190	145	167
55–64	412	242	325
65–74	920	532	723
75–84	1,883	1,338	1,590
85+	2,986	2,649	2,775

Source: Australian Institute of Health and Welfare. Stroke hospitalisations (principal diagnosis), by age and sex, 2016–17. AIHW analysis of the National Hospital Morbidity Database. 2017.

Table II. Comparison with other severity-based triaging tools

	**ACT-FAST**						**CPSSS**	**LAMS**	**RACE**	***Current practice****		
	**Time saving of 52 mins**	**Time saving of 40 mins**	**Time saving of 61.5 mins**	**Time saving of 30 mins**	**Time delay in tPA of 30 mins**	**Extended LVO definition**				***40%***	**25%**	**60%**
Sensitivity	0.826					0.758	0.877	0.222	0.845	-	-	-
Specificity	0.779					0.818	0.475	0.886	0.601	-	-	-
Total Costs (AUD)	$29,726	$29,746	$29,745	$29745	$29,740	$30,357	$29,631	$30,020	$29,651	$29,770	*$29787*	*$29746*
Total QALYs	5.005	5.001	5.008	4.996	5.001	5.008	4.991	4.986	4.993	*4.999*	*4.996*	*5.003*
Total LYs	5.3	5.2	5.3	5.2	5.2	5.3	5.2	5.2	5.2	5.2	5.2	5.3
Number of EVT^	0.072	0.072	0.072	0.072	0.072	0.112	0.071	0.073	0.072	*0.069*	*0.069*	*0.069*

Abbreviations: AUD: Australian dollars; QALY: quality-adjusted life year; LY: life year; LVO: large vessel occlusion; EVT: endovascular thrombectomy.
*varying the proportion of metropolitan patients being closest to the CSC (i.e. no need to bypass); ^ the average number of EVT performed.

Table III: results of using average time-saving in EVT from bypassing

	**ACT-FAST**	**Control**	**Difference**
Total costs (AUD)	$29,726	$29,770	*-$44*
Cost of acute hospitalization*	$14,297	$14,302	*-$5*
Cost of management	$14,140	$14,138	*-$2*
Cost of triaging	$12	$0	*$12*
Cost of ambulance transfer	$1,277	$1,330	*-$53*
Total QALYs	5.007	4.999	*0.008*
Total LYs	5.254	5.248	*0.006*
Number of EVT procedure	0.072	0.069	*0.003*
3-month functional outcome			
mRS≤2	70,779 (70.78%)	70,384 (70.38%)	
mRS>2	29,221 (29.22%)	29,616 (29.62%)	

**cost of acute hospitalization includes the costs related to the index hospitalization itself, thrombolysis, and endovascular thrombectomy where applicable.*

Online Figures

Figure I. Illustration of the simulation process

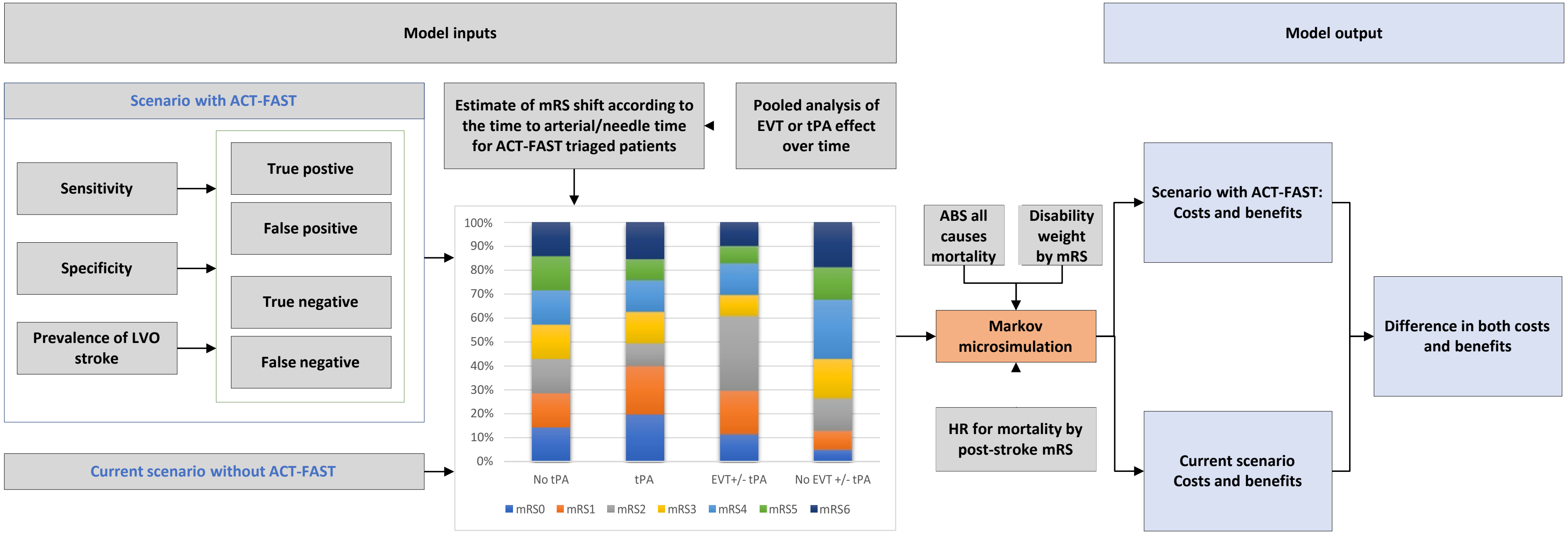

Figure II. Tornado diagram for one-way sensitivity analysis (vaying the diagnostic performance)

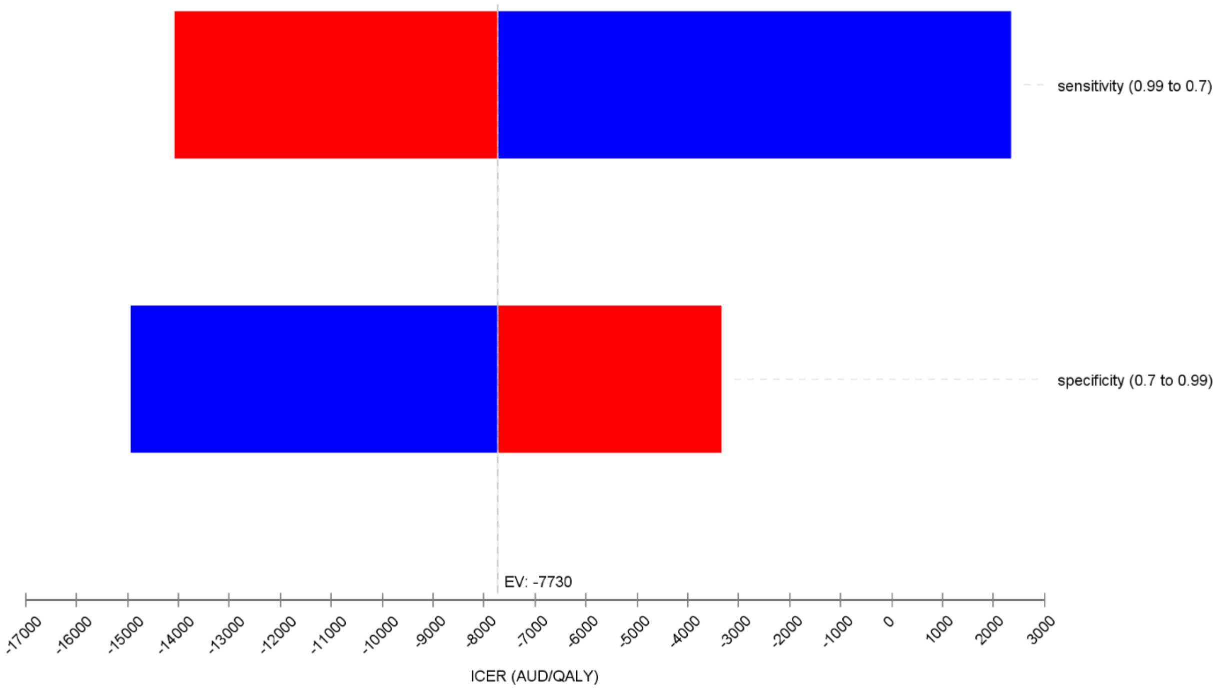

EV: expected value for the base case cost effectiveness analysis

ACT-FAST Algorithm (including the eligibility criteria for EVT–positive if all criteria met)

ARM, CHAT, TAP – after FAST/MASS assessment (ACT-FAST)

Algorithm for identifying severe stroke patients likely to require endovascular clot retrieval surgery

Step 1

☐

Proceed if YES (otherwise stop)

ARM (one sided arm weakness)

Position both arms at 45 degrees up from horizontal with elbows straightened and ask patient to hold rock steady. Vocally encourage the patient to hold up if arm begins to fall. The test may be repeated if unsure the first time.

Does just one arm fall completely to stretcher within 10 seconds of being held up?

In a patient that is ***uncooperative or not able to follow commands***, this item is positive if you clearly witness minimal or no movements in one arm and normal spontaneous movements in the other.

Answer no if both arms are similarly weak, or testing is clearly affected by shoulder problems or pain

Step 2

☐

Proceed if YES (otherwise stop)

If **RIGHT ARM** weak

CHAT (severe speech deficit)

Make an assessment from overall interaction and from your routine assessment. You may ask the patient to repeat a phrase or to perform simple tasks (eg making a fist, opening mouth).

Is there a severe language difficulty (not just slurring)?

- **Mute?**
- **Speaking gibberish/incomprehensible?**
- **Unable to follow simple commands?**

For ***non-English speakers***, use family/friends to translate and do not assume they are mute. If this is not possible, you may use a positive shoulder tap test (see IF LEFT ARM WEAK) instead to progress (in this scenario only).

If **LEFT ARM** weak

TAP (gaze & shoulder tap test)

1. Stand on the side that the patient is weak.
2. (Open eyelids if required) Observe if the patient has consistent and obvious gaze preference (of both eyes) away from the side of weakness, if so the test is ***positive*** - otherwise,
3. Tap the patient twice on the shoulder and call their first name in an audible voice – the test is ***positive*** if the patient does not quickly turn their head and eyes to fully focus on & notice you.

Proceed to next step if the test is positive.

This tests for severe gaze preference and hemi-neglect. It is acceptable to simply observe an obvious gaze preference (away from weak side) from the end of the stretcher.

Step 3

☐

IF YES: FOLLOW STUDY PROCEDURES FOR "ACT-FAST POSITIVE"

Any step negative = "ACT-FAST NEGATIVE"

ELIGIBILITY for endovascular clot retrieval
(All criteria must be met)

1. Deficits are NOT pre-existing (mild deficits that are now significantly worse are OK)
2. **Onset of symptoms <24 hours** - either witnessed, or time that patient was last known to be symptom-free (including wake-up)
3. Patient was **living at home independently** with only minor assistance - patient must be completely independent with hygiene/personal care tasks and walking (walking aids OK)
4. Patient has the following: (stroke mimic screen)
   - NOT comatose/near-comatose
   - NO history of seizure preceding symptoms
   - Blood glucose >2.8 mmol/l
   - NO definitely known (& active) malignant brain cancer
5. **No rapid improvement** of symptoms prior to loading to hospital (NEW CRITERIA)

Source: Figure from Zhao et al. Stroke, 2018; 49: 945-51.
